# Exercise and Reduced Nicotine Content Cigarettes in Adult Female Smokers: A Pilot Trial

**DOI:** 10.3390/ijerph19116647

**Published:** 2022-05-29

**Authors:** Chaofan Li, Nengliang Yao, Stephanie L. Miller, Christopher Macpherson, Taryn Hassinger, Kaitlin Love, Steven K. Malin

**Affiliations:** 1 Centre for Health Management and Policy Research, School of Public Health, Cheeloo College of Medicine, Shandong University, Jinan 250012, China; cfli@aging.org.cn (C.L.); ayao@aging.org.cn (N.Y.); 2 NHC Key Lab of Health Economics and Policy Research, Shandong University, Jinan 250012, China; 3 Division of General Medicine, Geriatrics & Palliative Care, University of Virginia, Charlottesville, VA 22904, USA; 4 Department of Kinesiology, University of Virginia, Charlottesville, VA 22904, USA; leighona.miller@gmail.com (S.L.M.); macphersoncr@mymail.vcu.edu (C.M.); 5 Department of Medicine, University of Virginia, Charlottesville, VA 22904, USA; teh3rz@virginia.edu; 6 Division of Endocrinology and Metabolism, University of Virginia, Charlottesville, VA 22904, USA; kml2w@virginia.edu; 7 Department of Kinesiology & Health, Rutgers University, New Brunswick, NJ 08901, USA; 8 Division of Endocrinology, Metabolism & Nutrition, Rutgers University, New Brunswick, NJ 08901, USA; 9 New Jersey Institute for Food, Nutrition and Health, Rutgers University, New Brunswick, NJ 08901, USA; 10 Institute of Translational Medicine and Science, Rutgers University, New Brunswick, NJ 08901, USA

**Keywords:** physical activity, smoking cessation, insulin resistance, obesity, cardiovascular disease

## Abstract

**Background:** Although Reduced Nicotine Cigarettes (RNC) are suggested to improve smoking cessation and cardiometabolic health in relation to cancer risk, the effectiveness of exercise training with RNC on smoking cessation and cardiometabolic health is unknown. **Methods:** Female smokers (N = 27) were randomized to: (1) usual nicotine cigarettes (i.e., control), (2) RNC or (3) RNC plus exercise treatment for 12 weeks. Smoking withdrawal symptoms (e.g., Wisconsin Smoking Withdrawal Scale) and cardiometabolic health (e.g., weight, VO_2_max, resting respiratory exchange ratio (RER), glucose, HOMA-IR) were examined before and after treatment. **Results:** Treatments had no differential effect on weight (*p* = 0.80; partial η^2^ = 0.29), VO_2_max (*p* = 0.20, partial η^2^ = 0.18), or total cholesterol/HDL ratios (*p* = 0.59, partial η^2^ = 0.06). However, RNC + Exercise tended to maintain RER (i.e., fat oxidation; *p* = 0.10, partial η^2^ = 0.10) as well as insulin resistance (*p* = 0.13, partial η^2^ = 0.25) and cortisol compared (*p* = 0.06, partial η^2^ = 0.30) with control and RNC. Increased VO_2_max was also associated with lower nicotine dependence scores (r = −0.50, *p* < 0.05). **Conclusion:** In this pilot study, improved fitness was associated with lower nicotine dependence. Additional work is warranted to examine the effects of exercise in smokers as a tool to improving smoking cessation and lower disease risk.

## 1. Introduction

While general smoking prevalence tends to decline globally, cigarette smoking induced mortality has increased in the United States over the last 50 years in men and women [1]. Tobacco smoking is thought to induce oxidative stress that dampens the immune system, promotes insulin resistance, and induces atherosclerosis [2]. This is consistent with prior work showing that smokers have greater inflammation (e.g., C-reactive protein (CRP)) and cortisol levels than non-smokers [2,3]. To reduce exposure to nicotine from cigarettes and thereby prevent disease risk related to cancer and cardiovascular disease, reduced nicotine cigarettes (RNC) have emerged containing between 0.3 to 8 (9 for menthol) mg of nicotine per cigarette versus the higher 11.4 mg in traditional cigarettes [4,5]. Indeed, RNC cigarettes decrease nicotine exposure, numbers of cigarettes smoked, and nicotine dependence [6]. This could be clinically relevant since smoking cessation profoundly reduces risk for cardiovascular disease (CVD), stroke, and cancers within as little as 1–5 years [7,8,9,10]. However, major barriers to smoking cessation are increased physiological and mental stress from nicotine withdrawal as well as 4–9 kg anticipated weight gain [11]. Thus, combating this stress from nicotine withdrawal is an important consideration for designing optimal programs that promote smoking cessation.

A possible approach towards supporting smoking cessation as well as combating disease risk in people who smoke is exercise. We have shown that exercise reduces body weight/fat and lowers insulin resistance as well as CVD risk through lowering adipose-derived inflammation [12,13,14]. Thus, it would be reasonable to expect that adding exercise to an RNC program may minimize stress responses and/or improve general cardiometabolic health. In fact, exercise training lowers mortality via raising aerobic fitness (i.e., VO_2_max,) and improves quality of life [15]. However, to date, few studies have been systematically designed to assess obesity related risk following smoking cessation [16,17] and/or exercise and smoking cessation [11,17,18]. In fact, a key gap in knowledge exists in the literature on the effectiveness of exercise training on smoking cessation symptoms during RNC use that contribute to more favorable cardiometabolic health and decreased cancer risk [19]. Hence, we tested the hypothesis that exercise would promote greater nicotine cessation success when combined with RNC compared with RNC and normal nicotine cigarettes with no exercise.

## 2. Materials and Methods

### 2.1. Eligibility and Recruitment

*Eligibility*: Female participants between 18–64 years of age with a BMI of 18–40 kg/m^2^ who were sedentary (<60 min/wk. of exercise) and smoked >4 cigarettes/day for at least a year who were not pregnant were included. Women alone, compared with mixed sexes, were recruited to improve weight homogeneity responses to exercise, given weight loss following exercise is generally less in women compared with men when matched on time of activity. Participants self-reporting use of weight altering medications (e.g., psychotropic drugs, insulin, phentermine, bupropion SR, etc., for the past 6 months), who experienced >2 kg weight change in past 3 months, or who had a medical condition (e.g., respiratory diseases, kidney, or liver diseases, etc.) were excluded.

*Recruitment*: Recruitment was performed via flyer distribution through Facebook and the local newspaper in rural counties around the University of Virginia between 2017–2019. Participants (see Table 1 for demographics) were randomly assigned to a control, RNC cigarette or a RNC + Exercise group (*see below for details*). 

*Screening*: All participants underwent a physical examination with EKG to ensure safety during exercise. Verbal and written informed consent was obtained prior to testing according to the University of Virginia Human Subjects IRB (IRB-HSR # 11219). 

### 2.2. Study Design

#### 2.2.1. Nicotine Protocol

##### Control Group

The control group smoked research cigarettes approximately matching the nicotine content of their preferred cigarette brand (about 11.4 mg). We provided them 6 weeks’ worth of cigarettes, and then mailed them the second 6-week intervention supply at week 5. However, following the 12-week assessment they were offered a one month gym membership to facilitate smoking cessation via exercise. Group adherence was assessed at end of study by the exercise physiologist and/or investigators by checking empty cigarette cartons. The three groups were not limited in terms of cigarette numbers smoked.

##### RNC with and without Exercise

The experimental group smoked RNC cigarettes (4 mg per cigarette). Participants were provided cigarettes in a similar manner to control. These research cigarettes were approved by and ordered from the U.S. FDA.

In addition to smoking RNC cigarettes during the intervention, some participants were randomized to exercises 3 d/wk. on a motorized treadmill at 75–85% of their respective heart rate max (HRmax) obtained from the VO_2_max test for 60 min/d under supervision of an exercise physiologist. Individuals were also instructed to exercise 2 d/wk. at 50–60% of their HRmax on non-supervised days to promote recovery and facilitate fitness adaptations. Participants were provided with heart rate monitors (Polar, Inc., Dayton, OH, USA) to gauge exercise intensity and kept a log of exercise intensity/duration on non-supervised days. Compliance was assessed by the exercise physiologist checking journals following the non-supervised exercise session. 

#### 2.2.2. Metabolic Control Protocol Prior to Assessment

Participants were instructed to consume ~200 g/d of carbohydrates and refrain from alcohol and vigorous non-exercise physical activity for at least 72 h before their clinical assessment. Participants were instructed to record food intake prior to pre-test measures. These same diaries were collected and then given back to participants with instruction to consume the same foods after the intervention for post-tests. Participants were also instructed to avoid dietary supplements, caffeine, and medications (e.g., antihistamines, anti-hypertensive medications, metformin, etc.) for 24 h before each study visit. Because all women included in the study were of child-bearing age, they were tested during the early follicular phase (days 2–8) of the menstrual cycle, based on communications, to minimize the impact of hormonal fluctuations on metabolic parameters. Participants were instructed not to not smoke cigarettes on the morning of and prior to urine/blood work. Participants randomized to exercise were also instructed to avoid strenuous exercise for at least 24 h prior to post-testing. Following an 8–12 h overnight fast, participants reported to the Clinical Research Unit (CRU) for metabolic testing between 0700–0900 eastern time.

#### 2.2.3. Outcome Variables Assessment

##### Body Fat, Fitness, and Metabolism

Weight, fat mass, and fat-free mass were measured by Bodpod (COSMED, Chicago, IL, USA) and waist circumference by plastic tape measures, respectively. VO_2_max was performed on the treadmill in the Exercise Physiology Core Laboratory using standard criteria (e.g., RER > 1.0, etc.). Resting metabolic rate (RMR) and respiratory exchange ratio (RER) were assessed using indirect calorimetry (V_max_ Encore, Viasys SensorMedics, Yorba Linda, CA, USA) as described previously by our group to assess metabolism [12,20,21]. 

##### Withdrawal Symptoms

Major nicotine withdrawal symptoms were assessed using the Wisconsin Smoking Withdrawal Scale, a 28-item scale [22]. These scales included anger, anxiety, sadness, concentration, hunger, somatic symptoms, sleep, and craving. The items were scored on a 5-point scale, with 0 indicating strongly disagree and 4 indicating strongly agree. This response scale allowed reverse-scored items to use the same response scale. 

*Smoking Cessation*: Changes in cigarettes per day were measured by self-reported cigarettes per day. Reduction in nicotine dependence was determined by the Fagerstrom Test. The test is a standard instrument for assessing the intensity of physical addiction to nicotine (a sample is attached) [23]. Changes in expired carbon monoxide (CO) were assessed by The Micro+™ Smokerlyzer^®^ CO monitor (Bedfont, England). These monitors measure CO in parts per million (ppm) in a breath. The breath CO level has been shown to have a close relationship with the level of CO in the blood known as carboxyhemoglobin (%COHb) [24]. Non-smokers have lower CO concentrations than smokers. 

Cotinine, anabasine, and nornicotine were also assessed via blood samples (see below) before and after treatment to assess nicotine metabolism. 

##### Plasma Cardiometabolic Health Measures

Fasting blood draws were collected to assess glucose, blood lipids (e.g., TG, HDL, LDL, total cholesterol (TC)), insulin, leptin, and cortisol levels. Insulin resistance was assessed by HOMA-IR (fasting insulin × fasting glucose/22.5). All blood was sent to the University of Virginia medical laboratory for testing using routine clinical assays. 

### 2.3. Statistical Analysis

Mean and standard deviation are used throughout. Skewness and kurtosis were calculated to test for normality. Analysis of variance (ANOVA) or Kruskal-Wallis H test was used to compare participant characteristics depending on normally vs. non-normally distributed data at baseline and post-test accordingly. A two-way repeated measures ANOVA was also used to assess treatment differences (treatment × time) in measured outcomes. For the non-normality outcome variables, log-transformation was used to perform two-way repeated ANOVA. Effect sizes were calculated using partial η^2^, with 0.01, 0.06, and 0.14 representing small, medium, and large effect sizes, respectively. Pearson or Spearman ranked correlations were performed to determine associations when appropriate. Data analysis was performed using SPSS 25.0. Significance was accepted as *p* < 0.05 and trends are discussed as *p* = 0.05 to *p* = 0.10. 

## 3. Results

### 3.1. Participant Characteristics

There were no significant differences in age, BMI, or CVD risk factors across groups before the intervention (Table 1). There were also no effects of RNC or RNC + Exercise on body weight/fat, although participants notably did not gain weight in either RNC groups or RNC + Exercise group trended towards reduced weight by 1 kg (partial η^2^ = 0.29). Surprisingly based on the supervised exercise dose, RNC and RNC + Exercise did not significantly increase VO_2_max despite a large effect size (*p* = 0.20, partial η^2^ = 0.18), with only RNC + Exercise having modest effects in maintaining RER (i.e., fat oxidation) compared with control and RNC (*p* = 0.10, partial η^2^ = 0.10).

### 3.2. Cardiometabolic Health

Insulin levels were maintained in the RNC + Exercise group, whereas they trended towards an increase, 5.8 uIU/mL and 4.6 uIU/mL in the RNC and control groups, respectively (main effect of treatment, *p* < 0.10, partial η^2^ = 0.25). Cortisol increased by 5.8 μg/dL in the control group (*p* < 0.05) and 2.4 μg/dL in the RNC group (*p* = 0.14), but was maintained at 11.0 μg/dL in the RNC + Exercise group (*p* < 0.06, partial η^2^ = 0.30). Although TC in the RNC + Exercise group was higher than that in the RNC group (main effect of time, *p* < 0.05, partial η^2^ = 0.23), there was no effects on TC/HDL ratios (*p* = 0.59, partial η^2^ = 0.06).

### 3.3. Smoking Cessation and Withdrawal Symptoms

**Figure 1:**
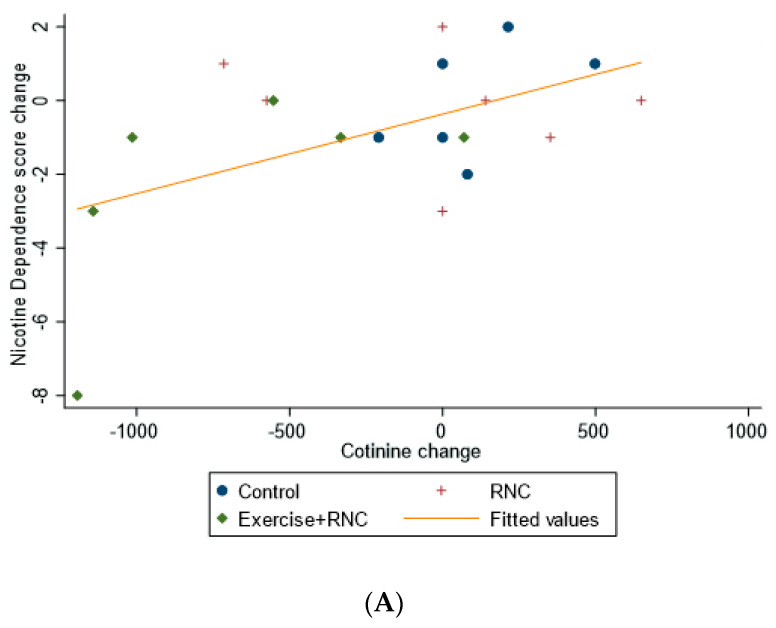

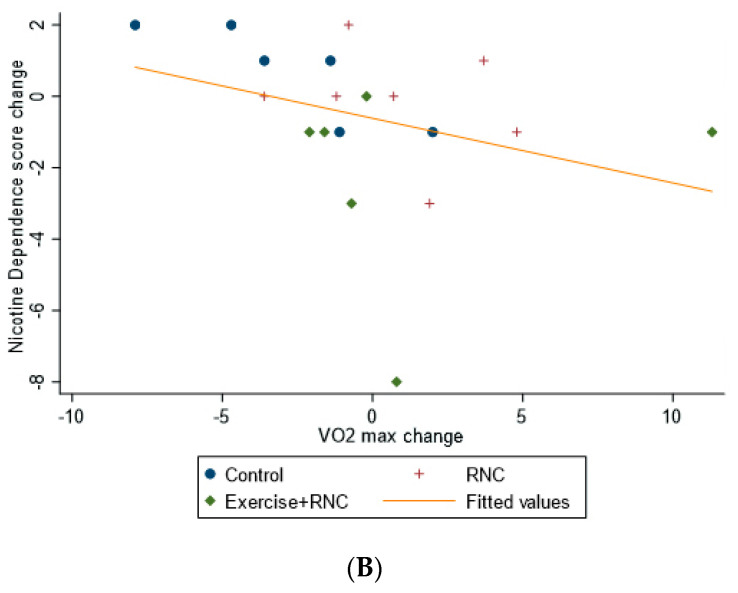
(**A**). Pearson correlation between nicotine dependence score change and cotinine change. Note: r = 0.52 (*p* = 0.02). (**B**). Spearman ranked correlation between nicotine dependence score change and VO_2_max change. Note: r = −0.50 (*p* = 0.03).

Cotinine levels in the control and RNC group increased, while they decreased in the RNC + Exercise group (main effect of treatment, *p* = 0.01, partial η^2^ = 0.43, Table 2). Interestingly, anabasine and nornicotine both followed similar patterns, whereby RNC + Exercise had medium to large effect on reducing plasma concentrations (partial η^2^ = 0.13 and partial η^2^ = 0.17, respectively) despite not reaching statistical significance (*p* = 0.38 and *p* = 0.37, respectively, Table 2). The nicotine dependence score in the RNC + Exercise group at week 12 trended lower than the other two groups with large effect sizes (*p* = 0.08, partial η^2^ = 0.25), and the reduction in this nicotine dependence was associated with lower cotinine (r = 0.52, *p* = 0.02) rises in VO_2_max (r = −0.50, *p* = 0.03; Figure 1).

## 4. Discussions

The primary finding of this pilot study was that increasing VO_2_max was significantly related to reductions in nicotine dependence. In turn, those individuals with lowered cotinine exposure also experienced declines in nicotine dependency. Interestingly, this lowered cotinine exposure was observed in those undergoing RNC + Exercise treatment only. This is somewhat surprising since prior work reported that users of RNC cigarettes changed smoking behavior and reduced nicotine exposure and dependence [4,25]. Although this could indicate more cigarettes smoked in the RNC group in the day prior to testing, we have no readily apparent reason to expect this based on self-reported compliance checks within our study between treatment groups or to suspect exaggerated cotinine effect with RNC alone. Since cotinine is a primary metabolite of nicotine, one speculation is that exercise fostered improved cotinine/nicotine metabolism, thereby leading towards reduced exposure. Indeed, nornicotine and anabasine also had medium to large effect sizes following RNC + Exercise. Few studies, however, have been systematically designed to assess fitness related smoking cessation and metabolic consequences [11,16,17,18]. In fact, these studies are limited in showing positive effects of exercise on smoking cessation because questionnaires versus structed exercise were used [11,16,17,18]. Therefore, our study extends on this previous work by showing that physical activity/exercise mediated fitness may be an adjunctive therapy to RNC that reinforces smoking cessation. 

A consideration of this pilot work is the benefit of adding aerobic exercise to a smoking cessation treatment option of RNC for cardiometabolic health. Nearly 33% of all smokers have some form a chronic disease, including stroke, type 2 diabetes, cardiovascular disease and cancer [8,26,27]. As such, using RNC cigarettes alone may have public health significance since regular smokers who switched to RNC cigarettes had greater decreases in nicotine exposure, numbers of cigarettes smoked, and nicotine dependence, although direct health effects are unclear [4]. Herein, we noted that RNC yielded no consistent effect in blood pressure, lipids, or glucose related outcomes. Although not statistically significant, it is of interest that the control group gained approximately 4 kg weight, while RNC maintained and RNC + Exercise decreased weight by about 1 kg. This corresponded with cravings having large effect size reductions following RNC + Exercise as well as RER elevations within the control and RNC groups, but preservation following RNC + Exercise. However, the implication is unclear since there were no differences in body fat despite about 1 kg weight los. In either case, when considering the addition of exercise to RNC, we noted a trend towards better circulating insulin profiles than either RNC or control. This finding highlights that exercise, independent of RNC, promotes the reduction of insulin resistance. These observations are consistent with prior studies by our group [28] and others [29,30] in non-smoking adults, reporting that exercise training is effective at promoting insulin action. 

Nicotine withdrawal can be uncomfortable and promote feelings of depression, having trouble sleeping, feeling irritable‚ having trouble thinking clearly, feeling restless, altering heart rate, and feeling hungrier. Aerobic exercise can improve mental health, control stress, reduce appetite, and increase RMR/fat utilization [19,31]. While we did not detect statistical differences in withdrawal related outcomes, it is likely that we are underpowered in this pilot study. Thus, these results provide preliminary work to power larger clinical studies on using RNC cigarettes and exercise to reduce smoking prevalence [4]. 

This study has limitations to consider. This pilot trial is exploratory in nature. Our focus was on descriptive statistics and estimation, rather than formal hypothesis testing [32]. Regardless, given that nicotine dependency scores were a key outcome for smoking cessation, and aerobic fitness served as an indication of exercise effectiveness, we estimated sample size estimates based on our findings. Using G*power software, with a partial η^2^ = 0.25, effect size = 0.577, power set at 0.80 and *p* = 0.05, it was estimated that 12 participants would be needed per group to detect statistical difference. Likewise, for fitness, with a partial η^2^ = 0.18, effect size = 0.468, power set at 0.80 and *p* = 0.05, it was estimated that 15 participants would be needed per group to detect statistical difference. This study also had only 27 participants and seven dropped out. The main reason for drop out related to time commitment for exercise. In turn, future exercise trials should consider telemedicine as a vehicle to promote physical activity. Furthermore, future studies could also conduct longer term follow-ups with multiple comparisons to understand fitness interactions with smoking cessation [33]. We selected 12 weeks as our prior exercise work [12] has shown this timeframe to raise fitness. Surprisingly, however, we didn’t detect statistical gains in fitness despite large effect sizes and people exercising under supervision for at least 3 out of 5 days a week at about 80% HRmax for 60 min/d. This dose, when combined with unsupervised exercise for 2 d/wk., resulted in exercise being performed for approximately 300 min/wk. It would seem unlikely that higher intensity or greater volumes would be required. Instead, we did not determine non-exercise activity in our participants and it is possible this dampened the effects of exercise on fitness gains. Thus, future work ought to consider non-exercise activity within physical activity interventions of smoking cessation. Another consideration is that we measured fasting insulin and calculated HOMA-IR only and did not use the euglycemic clamp to depict insulin resistance more accurately. We did not include an exercise + control cigarette group due to time and recruitment limitations although examining exercise added to routine cigarette is an important consideration for future study. Lastly, this pilot study included only female smokers, and future work should include males to enhance generalization. Still, women are understudied in cardiometabolic research and this provides valuable early data.

## 5. Conclusions

In conclusion, this pilot trial demonstrates the feasibility of using both exercise and RNC cigarettes in women. Our findings also show the potential to target gains in aerobic fitness as an approach to help lower nicotine dependence in female smokers. Further research is needed to follow up this work in a larger sample of women and men to determine the combined effects of RNC cigarettes and exercise on smoking cessation and cardiometabolic health.

## Figures and Tables

**Table 1:** Effect of RNC with or without Exercise on cardiometabolic health.

Outcome Variables	Control (N = 7)	RNC (N = 7)	RNC + Exercise (N = 6)	Time X Treatment ANOVA (*p*-Value)	Time X Treatment Effect Size (Partial η^2^)
**Demographics**					
Age	45.0 ± 12.0	38.6 ± 13.0	45.7 ± 7.9	0.80	0.03
**Body Fat, Fitness, and Metabolism**					
Weight (kg)				0.07	0.29
Baseline	74.7 ± 17.3	88.3 ± 12.8	81.2 ± 14.8		
12 Weeks	79.0 ± 16.0	88.5 ± 12.5	80.8 ± 15.4		
BMI (kg/m^2^)				0.51	0.09
Baseline	29.2 ± 6.4	32.6 ± 4.3	31.4 ± 6.9		
12 Weeks	29.7 ± 6.7	32.4 ± 4.1	31.2 ± 7.0		
Fat%				0.90	0.01
Baseline	38.1 ± 9.6	40.0 ± 5.2	43.7 ± 5.0		
12 Weeks	38.2 ± 10.6	39.5 ± 6.2	43.4 ± 4.3		
VO_2_max (mL/kg/min)				0.20	0.18
Baseline	26.8 ± 8.9	20.4 ± 4.7	22.5 ± 2.8		
12 Weeks	24.0 ± 8.4	21.2 ± 4.4	23.8 ± 4.7		
RMR (kcal/day)				0.54	0.08
Baseline	1344.8 ± 202.0	1483.3 ± 184.5	1302.0 ± 163.4		
12 Weeks	1362.3 ± 219.7	1479.7 ± 164.3	1301.2 ± 171.0		
RER				0.44	0.10
Baseline	0.83 ± 0.04	0.84 ± 0.05	0.82 ± 0.03		
12 Weeks	0.84 ± 0.05	0.89 ± 0.08	0.82 ± 0.05		
**Metabolic Health and Hormones:**					
Insulin (uIU/mL) **				0.13	0.25
Baseline	14.6 ± 8.0	9.8 ± 7.6	6.8 ± 2.2		
12 Weeks	19.2 ± 11.7 ^a^	15.6 ± 7.9 ^a^	6.3 ± 2.6		
Glucose (mg/dL)				0.50	0.10
Baseline	94.5 ± 11.7	93.7 ± 7.2	95.1 ± 9.3		
12 Weeks	102.8 ± 15.3	102.3 ± 17.4	94.3 ± 5.5		
HbA_1_C (%)				0.63	0.06
Baseline	5.4 ± 0.5	5.2 ± 0.3	5.5 ± 0.3		
12 Weeks	5.5 ± 0.6	5.2 ± 0.4	5.5 ± 0.3		
HOMA-IR *				0.32	0.19
Baseline	2.8 ± 1.2	2.2 ± 1.6	1.6 ± 0.7		
12 Weeks	4.3 ± 3.2	4.1 ± 2.7	1.6 ± 0.7		
Cortisol (μg/dL)				0.06	0.30
Baseline	10.7 ± 3.5	8.7 ± 3.2	11.3 ± 5.2		
12 Weeks	16.5 ± 3.7 ^b^	11.1 ± 2.9	11.0 ± 3.2		
hs-CRP (mg/L)				0.35	0.13
Baseline	3.0 ± 2.6	2.0 ± 1.7	6.3 ± 7.9		
12 Weeks	7.4 ± 9.2	1.6 ± 1.2	8.3 ± 11.2		
Leptin (ng/mL)				0.94	0.01
Baseline	18.5 ± 12.9	16.2 ± 9.6	16.5 ± 10.3		
12 Weeks	20.4 ± 13.9	18.0 ± 8.7	19.5 ± 9.5		
TC (mg/dL) **				0.12	0.23
Baseline	170.7 ± 24.2	176.4 ± 35.6	206.7 ± 30.7		
12 Weeks	175.3 ± 29.1	158.1 ± 27.4	208.7 ± 31.1		
TG (mg/dL)				0.09	0.26
Baseline	102.2 ± 80.0	123.4 ± 61.1	106.8 ± 79.1		
12 Weeks	134.8 ± 90.8	114.4 ± 51.0	110.3 ± 67.6		
HDL (mg/dL) **				0.44	0.10
Baseline	53.3 ± 7.9	43.1 ± 7.5	54.2 ± 13.7		
12 Weeks	55.3 ± 10.9	41.6 ± 6.3	56.8 ± 11.6		
LDL (mg/dL) *				0.35	0.12
Baseline	110.2 ± 14.0	112.9 ± 35.5	134.7 ± 23.7		
12 Weeks	97.5 ± 21.9	97.6 ± 28.2	133.7 ± 26.4		
TC/HDL				0.59	0.06
Baseline	3.3 ± 0.7	4.3 ± 1.4	4.0 ± 1.1		
12 Weeks	3.3 ± 0.9	3.9 ± 1.0	3.8 ± 0.9		

Note: Data are mean (SD), N = 20. There were no significant differences at baseline. * Main effect of treatment was significant at *p* < 0.10 level; ** Main effect of treatment was significant at *p* < 0.05 level. ^a^ Trending difference between baseline and 12-week post at *p* < 0.10 level; ^b^ Significant difference between baseline and 12-week post at *p* < 0.05 level; VO_2_max, insulin, hs-CRP and TG were log-transformed for analysis.

**Table 2:** Effect of RNC with and without Exercise on smoking withdrawal scale.

Outcome Variables	Control	RNC	RNC + Exercise	Time X Treatment ANOVA (*p*-Value)	Time X Treatment Effect Size (Partial η^2^)
**Withdrawal symptoms:**					
Wisconsin smoking withdrawal score				0.82	0.02
Baseline	1.9 ± 0.4	1.9 ± 0.6	1.9 ± 0.2		
12 Weeks	1.9 ± 0.5	2.1 ± 0.4	1.8 ± 0.5		
Anger				0.43	0.10
Baseline	2.5 ± 0.6	1.9 ± 0.9	1.7 ± 1.0		
12 Weeks	1.9 ± 0.8	2.2 ± 1.1	1.7 ± 0.8		
Anxiety				0.39	0.11
Baseline	2.4 ± 0.8	2.3 ± 1.0	2.5 ± 0.7		
12 Weeks	2.1 ± 0.8	2.5 ± 0.5	2.0 ± 0.8		
Concentration				0.79	0.03
Baseline	1.6 ± 1.3	2.0 ± 0.8	1.6 ± 0.4		
12 Weeks	1.5 ± 0.9	1.9 ± 0.6	1.3 ± 0.5		
Craving				0.07	0.27
Baseline	2.1 ± 0.7	1.9 ± 0.6	2.5 ± 0.6		
12 Weeks	2.0 ± 0.9	2.4 ± 0.4	1.8 ± 0.9		
Hunger				0.67	0.05
Baseline	2.2 ± 0.8	2.3 ± 1.0	1.6 ± 0.5		
12 Weeks	2.2 ± 1.0	2.6 ± 0.6	2.0 ± 0.9		
Sadness				0.98	0.003
Baseline	1.2 ± 0.4	1.5 ± 0.7	1.6 ± 0.6		
12 Weeks	1.4 ± 0.6	1.6 ± 0.9	1.9 ± 0.5		
Sleep				0.71	0.04
Baseline	1.9 ± 0.7	1.7 ± 0.9	1.7 ± 0.8		
12 Weeks	1.8 ± 0.9	1.5 ± 0.4	1.9 ± 1.0		
**Smoking Cessation Outcomes:**					
Nicotine Dependence score				0.08	0.25
Baseline	4.9 ± 1.9	5.4 ± 2.1	5.7 ± 2.5		
12 Weeks	5.1 ± 2.5	5.3 ± 1.7	3.3 ± 2.7		
Nicotine (ng/mL)				0.68	0.06
Baseline	636.2 ± 491.0	200.3 ± 179.8	462.6 ± 440.8		
12 Weeks	582.0 ± 500.5	305.8 ± 454.2	265.4 ± 523.2		
Nornicotine (ng/mL)				0.38	0.13
Baseline	62.9 ± 43.9	33.4 ± 16.5	51.4 ± 30.6		
12 Weeks	68.3 ± 48.3	42.7 ± 27.8	35.8 ± 47.9		
Cotinine (ng/mL)				0.01	0.43
Baseline	824.5 ± 379.2	899.1 ± 333.6	1058.2 ± 314.4		
12 Weeks	921.8 ± 484.3	877.9 ± 456.1	363.9 ± 471.1 ^a^		
Anabasine (ng/mL)				0.37	0.17
Baseline	11.8 ± 9.9	3.5 ± 1.5	8.4 ± 2.3		
12 Weeks	11.5 ± 6.9	6.4 ± 4.8	5.2 ± 7.7		
CO (ppm)				0.95	0.01
Baseline	1.8 ± 1.1	2.1 ± 1.5	2.0 ± 2.0		
12 Weeks	1.3 ± 1.7	1.3 ± 1.8	1.3 ± 0.7		

Note: Data are mean (SD), N = 20. There were no significant differences at baseline. ^a^ Trending difference between baseline and 12-week post test at *p* < 0.10 level.

## Data Availability

Data is available upon reasonable request to the corresponding author.
